# Poly(I:C) induces controlled release of IL-36γ from keratinocytes in the absence of cell death

**DOI:** 10.1007/s12026-015-8692-7

**Published:** 2015-09-25

**Authors:** Ali A. Rana, Alexandra V. Lucs, James DeVoti, Lionel Blanc, Julien Papoin, Rong Wu, Christopher J. Papayannakos, Allan Abramson, Vincent R. Bonagura, Bettie M. Steinberg

**Affiliations:** The Elmezzi Graduate School of Molecular Medicine, 350 Community Drive, Manhasset, NY 11030 USA; The Feinstein Institute for Medical Research, 350 Community Drive, Manhasset, NY 11030 USA; Department of Molecular Medicine, Hofstra North Shore LIJ School of Medicine, 350 Community Drive, Manhasset, NY 11030 USA; Department of Otolaryngology and Communication Disorders, Hofstra North Shore LIJ School of Medicine, Hearing and Speech Center, 430 Lakeville Road, New Hyde Park, NY 11040 USA; Division of Allergy and Immunology, Department of Pediatrics, Hofstra North Shore-LIJ School of Medicine, 865 Northern Blvd, Great Neck, NY 11021 USA

**Keywords:** IL-36γ, Cytokines, Keratinocytes, TLR3, Papilloma

## Abstract

The epithelium is part of an integrated immune system where cytokines, toll-like receptors and their ligands, and extracellular vesicles play a crucial role in initiating an innate immune response. IL-36γ is a pro-inflammatory member of the IL-1 family that is mainly expressed by epithelial cells, but regulation of its expression and release are only beginning to be understood. Previous studies reported that IL-36γ is abundant in recurrent respiratory papillomatosis, a rare but devastating disease caused by human papillomaviruses (HPV) types 6 and 11, in which papillomas recurrently grow in and block the airway. Despite the overexpression of IL-36γ, papilloma tissues show no evidence of inflammation, possibly due to suppression of its release by HPVs. We have used primary human foreskin keratinocytes as a model to study IL-36γ regulation in normal epithelial cells. Low doses of poly(I:C) mediate expression and release of IL-36γ without inducing the cell death reported by those using high doses. PKR, an enzyme required for inflammasome activation, does not contribute to controlled release of IL36γ. The keratinocytes secrete IL-36γ in two forms, soluble and in extracellular vesicles. We conclude that there are two separately regulated pathways for the controlled secretion of IL-36γ from keratinocytes, which could contribute to the modulation of both local and systemic immune responses to viruses and other pathogens.

## Introduction

Recurrent respiratory papillomatosis (RRP) is a rare but devastating disease in which papillomas recurrently grow in and block the airway. RRP is caused by human papillomaviruses (HPVs), primarily types 6 and 11 [1, 2]. Papillomas are benign stratified squamous epithelial tumors characterized by a hyperplastic suprabasal epithelium surrounding cords of connective tissues [3].

The epithelium is part of an integrated immune system where cytokines, toll-like receptors (TLRs) and extracellular vesicles (EVs) play a crucial role in initiating innate immune responses. Anomalies within this system can lead toward disease. Patients with RRP respond to HPV antigens with a blunted adaptive immune response that is biased toward a T_H_2-like phenotype [4–6]. This bias appears to reflect an underlying innate defect [7]. However, when DeVoti et al. [4] analyzed the transcriptional profile of matched sets of papilloma tissues and normal airway tissues from the same RRP patients, *IL-36γ* was the gene that was most consistently elevated in the papillomas. IL-36γ is a pro-inflammatory member of the IL-1 family, made by keratinocytes in response to multiple stimuli [8, 9]. It is highly expressed in psoriasis, an immune-mediated inflammatory skin disease [10]. Despite its clear inflammatory potential, there is no evidence for inflammation in papilloma tissues. Resolving this paradox first requires a better understanding of the regulation of IL-36γ expression and release in normal epithelial cells, which is still quite limited [11]. In this study, we have used foreskin keratinocytes, which are also stratified squamous epithelial cells, as a model system to study the normal process.

Because IL-36γ lacks a signal sequence, it is not directed to the endoplasmic reticulum for secretion [11, 12]. Several nonclassical pathways of IL-1β secretion have been reported, including the inflammasome, secretory lysosomes, and various extracellular vesicles (EVs) [13]. Lian et al. [14] reported that high concentrations of the toll-like receptor 3 agonist poly(I:C), an analog of double-stranded RNA, induce IL-36γ expression and release through inflammasome-mediated pyroptosis. We have asked whether keratinocytes might be induced to express, and possibly release, IL-36γ through a different pathway that does not involve cell death. Such alternate regulation might explain the lack of evident inflammation in papilloma tissues.

## Materials and methods

### Cell culture and reagents

Neonatal human foreskins were obtained anonymously as surgical discards. The North Shore-LIJ Institutional Review Board determined that the study was exempt. Keratinocyte cultures were established as described [15], pooled, and expanded for no more than four passages on mitomycin C-treated J2-3T3 feeder cells in E-media [16]. Cell viability was assessed by trypan blue exclusion and by lactic dehydrogenase release as described [17]. For analysis of EVs, cells were cultured in medium supplemented with serum depleted of EVs as previously described [18]. For in vitro stimulation and inhibition assays, the following substances were used, at the concentrations shown and times indicated in the text: poly(I:C) (Invivogen, San Diego, CA), 2AP, 7DG, EGFR inhibitor (PD153035), MEK inhibitor (PD98059), p38 inhibitor (SB202190), PI-3 K inhibitor (LY294002) and JNK inhibitor (SP600125) (all from Sigma, Saint Louis, Missouri). For all experiments, the solvent for the specific reagent was used as a control. Experiments were done at least three times unless otherwise noted.

### Western blot analysis

Proteins were extracted as previously described [19]. IL-36γ levels were normalized to β-actin and expressed relative to controls treated with solvent. Primary antibodies were anti-IL-36γ at 1:400 (R&D system, Minneapolis, MN), anti-TSG101 at 1:2000 (Abcam, Cambridge, MA), and anti-β actin at 1:5000 (Sigma, Saint Louis, MO). LI-COR secondary antibodies were used at 1:500 for quantification by the Odyssey infrared imaging system (LI-COR, Lincoln, NE).

### Measurement of cytokine release

Keratinocyte-conditioned medium was analyzed by ELISA for accumulation of released IL-36γ. Studies in Figs. 2 and 3 were done with a kit from Aviscera Bioscience (Santa Clara, CA), and those in Fig. 4 were done using a kit from Sigma, Saint Louis, Missouri because there were problems with the Avicera plates. Results were initially measured as pg/ml and then normalized to the protein concentration in the cell monolayer releasing the cytokine. IL-1β ELISA was done using kits from R&D System (Minneapolis, MN). All studies were done as per manufacturers’ directions. EVs were isolated from conditioned medium as previously described [18] and analyzed by western blot.

### Statistical analysis

Statistical analyses were performed with a one-way analysis of variance (ANOVA) with Tukey–Kramer multiple comparisons test. *P* values <0.05 were considered significant; *p* values <0.001 were considered highly significant.

## Results

**Fig. 1:**
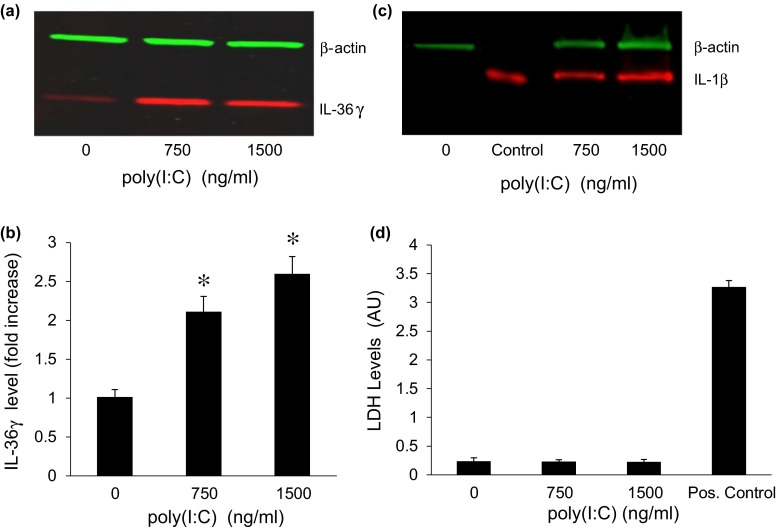
Low doses of poly(I:C) induce IL-36γ expression. **a** Representative western blot showing IL-36γ expression in keratinocytes stimulated with increasing concentrations of poly(I:C) for 48 h. **b** Quantification of intracellular IL-36γ. *Bars* show mean ± SD, relative to controls treated with vehicle (*n* = 6 experiments, *p < 0.001). **c** Representative western blot of keratinocytes treated with vehicle or with poly(I:C) for 48 h. Recombinant IL-1β at 20 ng/lane was used as a positive control. β-actin was used as a loading control. **d** Cells were treated with increasing concentrations of poly(I:C) for 96 h and the culture medium assayed for released lactic dehydrogenase (LDH) as a measure of cell death. Culture medium from cells lysed with the detergent NP40 served as a positive control

We first determined that poly(I:C) could induce IL-36γ expression in a dose-dependent manner (Fig. 1a, b), at concentrations lower than those used by Lian et al. [14] which causes subsequent cell death. IL-1β, the prototypical member of the IL-1 family of which IL-36γ is a member, was also induced by poly(I:C) (Fig. 1c). To rule out the possibility that the lower concentrations of poly(I:C) induced death of some of the cells, we used both trypan blue staining and assay of lactate dehydrogenase (LDH) in the culture medium. There was no detectable cell death as measured by trypan blue staining (not shown) or by LDH release (Fig. 1d). We were also unable to detect any IL-1β in the conditioned medium using a highly sensitive ELISA assay (data not shown), further evidence for the absence of any cell death.

**Fig. 2:**
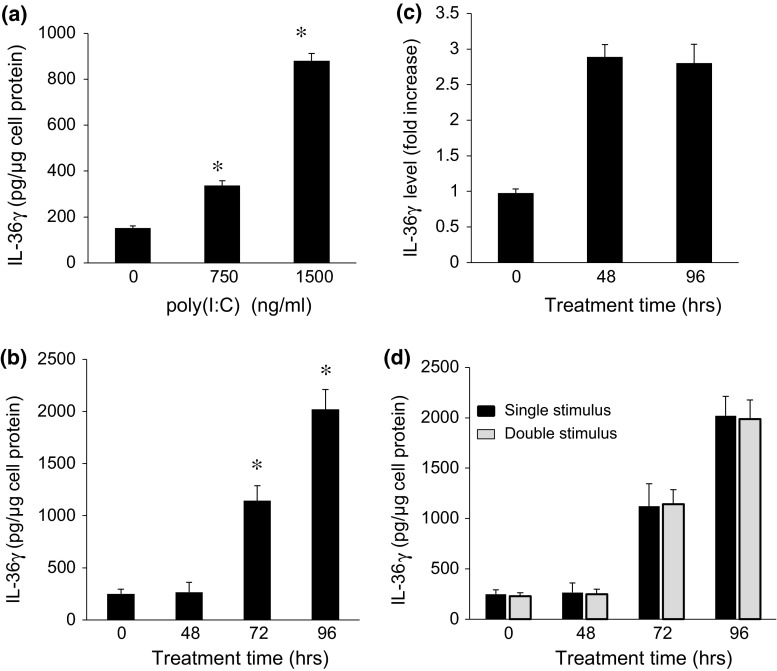
Low doses of poly(I:C) induce IL-36γ release in a dose and time-dependent manner. **a** Cells were treated with increasing concentrations of poly(I:C) for 96 h, and the conditioned medium analyzed by ELISA for accumulated IL-36γ. *Bars* show mean ± SD of IL-36γ per μg of cellular protein in the secreting monolayer (*n* = 8 experiments, *p < 0.001 relative to control cells treated with solvent). **b** Cells were treated with 1500 ng/ml of poly(I:C) and conditioned medium analyzed by ELISA at varying times. *Bars* show mean ± SD of IL-36γ levels, normalized per μg of cellular protein in the secreting monolayer (*n* = 4 experiments, *p < 0.001 compared to 0 time). **c**/**d** Cells were treated with 1500 ng/ml of poly(I:C) for 48 h, the medium removed, the cells washed, and medium replaced for an additional 48 h ± additional poly(I:C). Intracellular IL-36γ was measured by western blot (**c**) and IL-36γ levels in the conditioned medium measured by ELISA (**d**). *Bars* show mean ± SD of 4 experiments

Release of soluble IL-36γ was induced by these same lower concentrations of poly(I:C) (Fig. 2a). Soluble IL-36γ that accumulated in the medium could only be reliably detected at 72 h after treatment and was much greater at 96 h (Fig. 2b), although intracellular IL-36γ was markedly elevated at 48 h. To further analyze the relationship between kinetics of expression and release, the culture medium was changed at 48 hours, with and without additional poly(I:C), and IL-36γ measured at 96 h post-initial treatment. The additional 48 h of treatment with poly(I:C) had no effect on intracellular IL-36γ levels (Fig. 2c). Moreover, levels of IL-36γ were comparable whether poly(I:C) was added for only the first 48 h or was re-added for the subsequent 48 h (Fig. 2d). Together, these results suggest that the process of release requires an extended period of time after initiation, rather than simply being dependent on accumulation of intracellular IL-36γ, and the process does not require continuous stimulation with poly(I:C) once initiated.

**Fig. 3:**
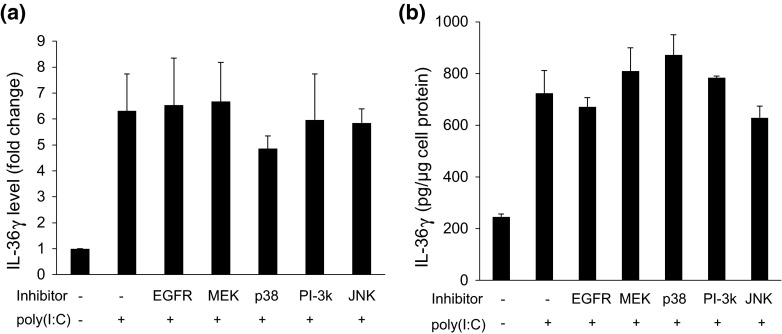
MAP kinases do not mediate the expression or release of IL-36γ in keratinocytes treated with low doses of poly(I:C). **a** HFKs were treated with vehicle (control) or with inhibitors of the EGFR (1 µM), MEK (50 µM), p38 (10 µM), PI-3 K (25 µM) or JNK (10 µM) for 3 h and then poly(I:C) (1500 ng/ml) was added for an additional 48 h and IL-36γ analyzed by western blot. IL-36γ levels were normalized to β-actin to correct for protein loading. *Bars* show mean ± SD, relative to controls (*n* = 8 experiments). **b** HFKs were pretreated with vehicle or inhibitor as in (**a**) for 3 h. Poly(I:C) (1500 ng/ml) was added as indicated, cells incubated for 96 h, and conditioned medium analyzed by ELISA for accumulated IL-36γ. *Bars* show mean ± SD of IL-36γ normalized to μg of cellular protein in the secreting monolayer, relative to cells treated with poly(I:C) in the absence of inhibitor (*n* = 8 experiments)

Respiratory papilloma cells constitutively express both activated MAP kinases [20, 21] and IL36γ [7], and TLR3 activation can activate MAP kinases in multiple cell types including keratinocytes [22–25]. We therefore asked whether activation of MAP kinases was required for the low dose of poly(I:C)-induced expression or release of IL-36γ (Fig. 3a, b) in normal keratinocytes. Surprisingly, inhibiting each of the MAPKs had no significant effect, although there appeared to be a very modest reduction in IL-36γ expression with the p38 MAP kinase inhibitor. Western blots confirmed that ERK activation was inhibited by the MEK inhibitor; there was no measurable activation of p38 MAP kinase with poly(I:C) and thus no detectable inhibition (data not shown).

**Fig. 4:**
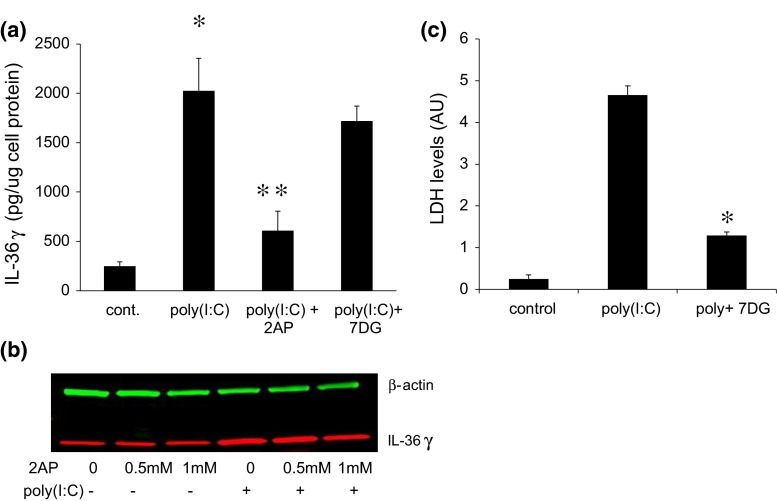
2AP suppresses poly(I:C)-induced IL-36γ release, while 7DG has no effect on release. **a** Cells were stimulated for 96 h with 1500 ng/ml poly(I:C) ± the broad spectrum PKR inhibitor 2AP (1 mM) or the more specific inhibitor 7DG (5 μM), and IL-36γ accumulation in the conditioned medium measured by ELISA.* Bars* show mean ± SD of 5 experiments (*p < 0.001 compared to control cells treated with solvent, **p < 0.001 compared to cells treated with poly(I:C) but no inhibitor). **b** Cells were treated for 96 h with 1500 ng/ml poly(I:C) ± 2AP at the concentrations shown, and intracellular IL-36γ levels determined by western blot with β-actin as a loading control. A representative blot is shown. **c** Cells were treated with 2 μM poly(I:C) ± 5 μM 7DG for 96 h and the culture medium assayed for released lactic dehydrogenase (LDH) as a measure of cell death. 7DG suppressed the elevated cell death induced by high-dose poly(I:C) (*p < 0.001)

Double-stranded RNA-dependent protein kinase (PKR) regulates inflammatory responses by activating multiple pathways, including the inflammasome [26], and PKR can be directly activated by poly(I:C) [27]. IL-36γ release was inhibited by the kinase inhibitor 2AP, which is frequently used as a PKR inhibitor, but not by the more selective PKR inhibitor 7DG [28] (Fig. 4a). To confirm that 2AP was inhibiting release and not simply affecting the expression of IL-36γ, we analyzed intracellular levels of IL-36γ by western blot. There was no effect of 2AP on either baseline or poly(I:C)-stimulated expression (Fig. 4b). We also considered the possibility that the dose of 7DG was insufficient to block PKR activation. 7DG significantly inhibited inflammasome-mediated cell death induced by a higher concentration of poly(I:C), as measured by LDH release (Fig. 4c). These results indicate that the controlled release of IL-36γ does not require activation of PKR or the inflammasome, and suggest that one of the many other kinases inhibited by 2AP may mediate release of soluble IL-36γ [29].

**Fig. 5:**
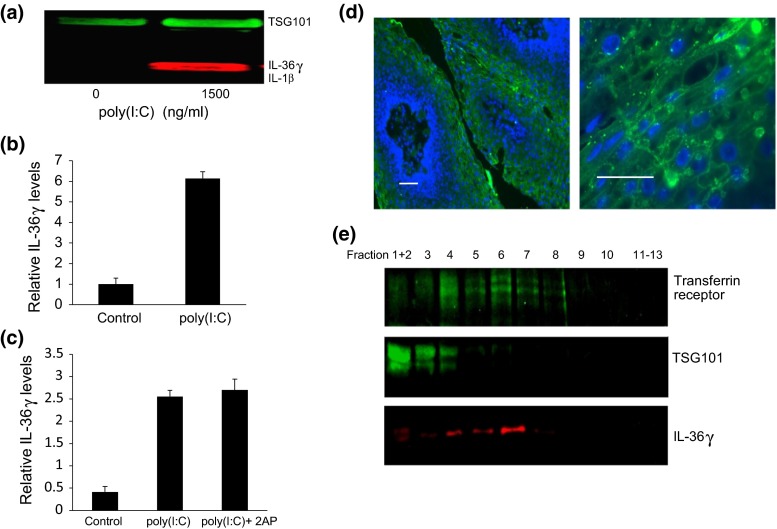
Poly(I:C) induces IL-36γ release in multiple extracellular vesicles (EVs), consistent with punctuate appearance of IL-36γ in suprabasal layers of papillomas. **a** Representative western blot. HFKs were stimulated with 1500 ng/ml poly(I:C) for 96 h, vesicles isolated by differential centrifugation, and analyzed by western blot using TSG101 as a marker for EVs and as a loading control. **b** Quantification of IL-36γ within vesicles. *Bars* show mean ± SD, relative to controls without poly(I:C) (*n* = 4 experiments, *p < 0.001). **c** Cells were treated with poly(I:C) ± 1 mM 2AP, and EVs isolated and analyzed as in “a.” *Bars* show mean ± SD, relative to controls of 4 experiments. **d** Sections of paraffin-imbedded papilloma tissues were incubated with goat anti-IL-36γ and visualized with fluorescein-conjugated donkey anti-goat IgG. DAPI staining of DNA was used as a counterstain. Images show representative papilloma sections. Bars = 40 µm. **e** Cells were stimulated with 1500 ng/ml poly(I:C) for 96 h, extracellular vesicles were extracted by differential centrifugation, separated on sucrose gradients and fractions from the gradients analyzed by western blot. TSG101 is a marker for endosomes and the transferrin receptor marks exosomes. A representative blot is shown

Cells secrete EVs that can mediate cell–cell communication through exchange of both protein and RNA [30]. We therefore asked whether IL-36γ can be secreted within EVs as well as in soluble form. Keratinocytes constitutively secreted EVs, independently of poly(I:C) stimulation, as demonstrated by the detection of TSG101 in EVs from the untreated controls. However, EVs from cells treated with poly(I:C) contained significantly more IL-36γ (Fig. 5a, b). The EVs did not contain detectable IL-1β, suggesting that incorporation of IL-36γ was specific. Unlike the release of soluble IL-36γ, secretion within EVs was not sensitive to 2AP (Fig. 5c), suggesting that these two processes are regulated separately. Consistent with the finding that IL-36γ is within vesicles, immunohistochemical staining of papilloma tissues showed that IL-36γ was primarily punctuated in appearance, and localized adjacent to the cytoplasmic and nuclear membranes of cells in the spinous layer (Fig. 5d). The level of expression in normal epithelial tissue was too low to be detected (not shown).

Different cell types have been shown to release different types of EVs, such as exosomes, ectosomes and microvesicles [31]. To analyze the type of keratinocyte EVs that contained IL-36γ, we isolated the vesicles, separated them on sucrose gradients, and performed western blots on each fraction (Fig. 5e). We used TSG101 as an endosomal marker and the transferrin receptor as an exosomal marker. TSG101 is a major component protein of the ESCRT machinery for exosome synthesis [32]. The transferrin receptor is a membrane carrier protein and was the first transmembrane protein found to be released in exosomes [33]. TSG101 was present in fractions 1–4 (between 1.000 and 1.042 g/ml), and the transferrin receptor was detectable in fractions 4–8 (between 1.042 and 1.180 g/ml). Exosomes usually float at a density between 1.08 and 1.18 g/ml [34]. We detected IL-36γ mainly in fractions 4, 5 and especially 6 (Fig. 5c), with lesser amounts in fractions 1–3, suggesting that IL-36γ is secreted in multiple different vesicles, but mainly associated with exosomes.

## Discussion

Keratinocytes are a key component of the innate immune response, reacting to infection by bacteria or viruses with expression of proinflammatory cytokines and chemokines that activate dendritic cells and Langerhans cells. Poly(I:C) has been shown to induced cytokine expression in keratinocytes through activation of both TLR3 and RIG-1, with the dsRNA-sensing kinase PKR playing a key role in the signaling downstream of both receptors [35]. Furthermore, poly(I:C) has been shown to induce expression of the proinflammatory cytokine IL-36γ in keratinocytes and induce cytokine release through pyroptosis, a form of inflammasome-mediated cell death [14]. PKR played an essential role in both processes, and studies have reported that PKR is required for inflammasome activation [28]. Finally, Karim et al. [36] reported that HPV16 suppressed poly(I:C)-induced expression of a number of proinflammatory genes, including components of the inflammasome, proposing that this was a mechanism for immune evasion by HPVs. However, all of those studies used high concentrations of poly(I:C), which are lethal to keratinocytes. Because massive cell death is not seen in conjunction with HPV infection, we set out to determine whether poly(I:C) could induce the expression and release of IL-36γ at sublethal concentrations.

In this study, we have shown that a low dose of poly(I:C) induces expression and release of soluble IL-36γ in a PKR-independent process that causes no measurable cell death. We estimate that approximately 5 % of the total intracellular IL-36γ accumulates in the culture medium. This could well be an underestimate of actual secretion if some of the IL-36γ is degraded or binds to and is taken back up by the keratinocytes. Future studies will be needed to determine the pathway(s) that mediate expression, but clearly they do not involve MAP kinase activation. This is consistent with the report by Yu et al. [37] that poly(I:C)-induced shedding of TNFR1 from airway epithelial cells is independent of MEK, Erk, JNK and Akt. The protracted period of time required for controlled release of soluble IL-36γ in our study was unexpected. Clearly, it did not reflect a delay in transcription or translation since we were able to detect high levels of intracellular IL-36γ 24 hours after poly(I:C) stimulation. Rather, we speculate that it reflects the time required to induce and possibly assemble a secretory mechanism that is yet to be determined.

We have also shown for the first time that IL-36γ is released from keratinocytes in multiple extracellular vesicles in response to poly(I:C) stimulation. Vesicular release is independent of PKR, and also independent of the unidentified kinase inhibited by 2AP that regulates soluble IL-36γ release. Our knowledge of the biogenesis and secretion of EVs has grown extensively in the last decade. The endosomal sorting complex required for transport is composed of four multi-protein complexes, ESCRT-0, 1, 2 and 3 and each has its own function in the biogenesis of EVs [38]. There is significant variation in ESCRT composition and function in different cell types [39], and this process has not been extensively studied in normal keratinocytes. However, studies in other cell types have shown that the determination of cargo within EVs is controlled and not random [32, 40, 41]. We have shown that EVs released from poly(I:C)-stimulated keratinocytes contain IL-36γ but not IL-1β, suggesting an active sorting process in response to poly(I:C) stimulation.

The ability of viable keratinocytes to release IL-36γ in both a soluble and an EV-encapsulated manner would suggest that it should be released by papilloma cells since they contain high intracellular levels, thus activating an inflammatory response in the larynx of RRP patients. The absence of inflammation, coupled with our finding that release of soluble IL-36γ is markedly delayed, suggests that release of this cytokine is an active and regulated process. HPV11 can suppress the release of soluble IL-36γ [7], and Honegger et al. [42] reported that HPV18 suppresses secretion of extracellular vesicles from HeLa cells. Further studies will be needed to define the molecular mechanism(s), whereby HPVs suppress the controlled release of cytokines from keratinocytes.

We conclude that there are two separately regulated pathways for the controlled release of IL-36γ from keratinocytes, which might have different biological activity. Future studies will be needed to characterize these pathways in detail and determine their role(s) in the modulation of both local and systemic immune responses to infection.
